# Minimally Invasive Surgical Technique for the Extraperitoneal Fixation of Acetabulum Fracture: Technical Feasibility Study in Cadaver

**DOI:** 10.1155/aort/2914086

**Published:** 2025-03-26

**Authors:** Piia Suomalainen, Essi Honkonen, Sami Nurmi, Anu Välikoski, Antti Siiki

**Affiliations:** ^1^Department of Orthopaedics and Traumatology, Tampere University Hospital, Tampere, Finland; ^2^Department of Gastroenterology and Alimentary Tract Surgery, Tampere University Hospital, Tampere, Finland

## Abstract

**Background and Objectives:** When operating on acetabular fractures in conventional open surgery, visualization of crucial structures can be challenging. In recent years there have been several case reports on laparoscopy-assisted acetabulum surgery in the literature. Therefore, we have developed this method further using extraperitoneal endoscopy to manage acetabulum fractures.

**Methods:** Operative technique: An experienced hernia surgeon familiar with the totally extraperitoneal laparoscopic technique facilitates access to the acetabulum area so that orthopaedic surgeons can focus on fixing the area with a plate and screws through laparoscopy ports.

**Results:** We developed this operative technique in a cadaver laboratory where we could easily fix and plate the acetabulum area with extraperitoneal endoscopy visualization in seven cadavers both on the left and right sides.

**Conclusions:** A minimally invasive full endoscopic procedure for acetabular fractures offers significant benefits over traditional open surgery due to faster rehabilitation, potentially less blood loss, and fewer wound complications. According to our initial experiences with cadavers, this minimally invasive method appears promising in terms of superior visibility and easier access to the otherwise narrow and difficult fracture site in the pelvic region compared to open surgery. Furthermore, this minimally invasive method seems feasible for exact plate placement under combined endoscopic and fluoroscopic visual control. The usefulness of this novel method in the minimally invasive treatment of acetabular fractures in real life, especially considering the practicality of proper fracture reduction, should be confirmed in future clinical trials.

## 1. Introduction

Acetabular fractures are rare in Western countries [1]. In the older population, the trauma mechanism is usually a fall from the same level, whereas high-energy trauma, such as motor vehicle accidents and falls from heights, are more common in the younger population [1]. For high-energy displaced acetabular fractures, operative treatment is the gold standard, and open reduction and internal fixation (ORIF) are traditionally used to manage these fractures. A standard approach to the anterior part of acetabular fractures has been the modified Stoppa approach, where the acetabulum is exposed through a low midline incision in the extraperitoneal region [2].

The problem with this approach is the visualization of the highest proportion of the pelvic brim, which is typically the highest point of the fracture. The peritoneum and the femoral vein and artery are located nearby and sometimes prevent the proper approach, reduction and plating of the fracture from being performed safely. In addition, some patients also have postoperative problems with the incision, including pain in the anterior part of the pelvis. Dissection and sometimes detachment of the rectus abdominis muscles can also cause serious postoperative morbidity.

To date, only a few clinical case reports have been published on methods similar to the one we are introducing in the present cadaver study. In a report from China, an endoscopic approach with fracture management was used using a 3D-printed model of the patient's pelvis. The plate for fixation was prebent according to the 3D model [3]. A case report from Germany introduces a technique similar to ours. However, an additional ilioinguinal lateral approach was used to reduce the fracture and introduce the plate. The authors of the report proposed that the method should become a standard procedure in the management of acetabular fractures in the future [4]. Mauffrey et al. reported a case report, where they conducted the procedure with the aid of fluoroscopy and endoscopy assistance. They fixed the fracture with percutaneous screws in contrast to our technique (plate) [5].

Studies in a cadaver setting have also been published. Kuper et al. published a report from a cadaver laboratory in which the technique used was similar to ours, except that we operate in the extraperitoneal space, and their technique is performed in the intraperitoneal space [6]. The same group has also published a technique to fix symphysis endoscopically in the extraperitoneal space and speculated that this technique can be further extended to reach the quadrilateral and acetabular area [7]. They have also published a clinical series of seven patients treated with endoscopic anterior pelvic plating with good results [8]. Hartel et al. conducted a series of endoscopic extraperitoneal cadaver operations, where they tested the hypothesis that the plating can be done in endoscopic manner. They concluded that new, longer instruments are mandatory to develop, but it can basically be done [9]. The technique of Trulson et al. is the most similar to ours, since they used a totally extraperitoneal endoscopic approach in a cadaver setting to reach the acetabulum area and fixed it with a plate [10].

In contrast to previous reports on endoscopic acetabulum surgery, in our setting, we have an experienced hernia surgeon who is familiar with the complete extraperitoneal endoscopy technique to facilitate access to the operative field, so that orthopaedic surgeons can focus on reduction and fixation of the fracture. We have identified this as a key point when making an exact and quick visualization of different anatomical structures from a new perspective. In fact, it is a widely known fact that patient safety is superior when you can concentrate on what you do best.

Due to the problems with the modified Stoppa approach outlined above, we have developed a novel technique where visibility is acquired using the endoscopic technique. Today, extraperitoneal endoscopy is commonly used in inguinal hernia surgery. Acetabular fracture surgery is performed in the same area as hernia surgery, so the approach itself is not new, but the idea that fracture can be treated entirely extraperitoneally is [11]. This study was conducted at the Tampere Surgical Education Center with seven cadavers, in which we operated on both the left and right sides. The Ethics Committee of the University of Tampere, Finland, approved the scientific use of the cadavers. In the study, cadavers with intact pelvic brim were operated on. No written consent has been obtained from the patients as there is no patient identifiable data included.

## 2. Methods

### 2.1. Operative Technique in a Cadaver Setting

**Figure 1:**
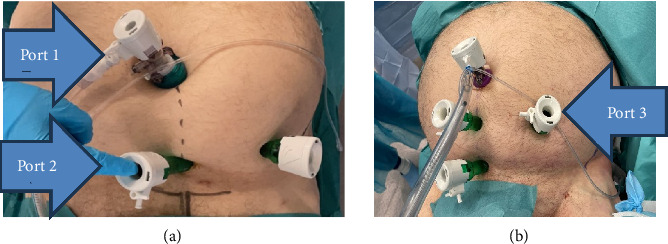
The placement of the 15 mm ports (a). An additional port can be placed between ports 1 and 2, if needed (b).

First, the patient is placed in a supine position on the operating table, ensuring free use of the O-arm or the C-arm. After a short infraumbilical incision is made at the midline, subcutaneous tissue is retracted to reveal the fascia of the rectus abdominis. The fascia is then incised and the rectus abdominis muscle is retracted laterally with a Langenbeck retractor. A 12 mm balloon endoscopic port ((Kii Sleeve 12/100 mm CFS22 × 1 with inflatable balloon, Applied Medical, CA, USA)) (port 1) is introduced into the extraperitoneal space and guided downward to the symphysis. The balloon is then inflated and then the 30° laparoscope (Endoeye Olympus Europe, Hamburg, Germany) is placed and a pneumopreperitoneum with 15 mmHg carbon dioxide insufflation is established. The correct position of the additional 15 mm port ((Kii Optical Access System 15 × 100 mm)) (port 2) just proximal to the symphysis is first probed with a needle in endoscopic visual control and the 15 mm port is inserted. An additional 15 mm port (port 3) is also inserted halfway between the midline and the anterior superior iliac spine (Figure 1). If the patient is very obese, an additional 15 mm port (port 4) can be placed just proximal to port 2 (Figure 1) to help maintain visualization.

**Figure 2:**
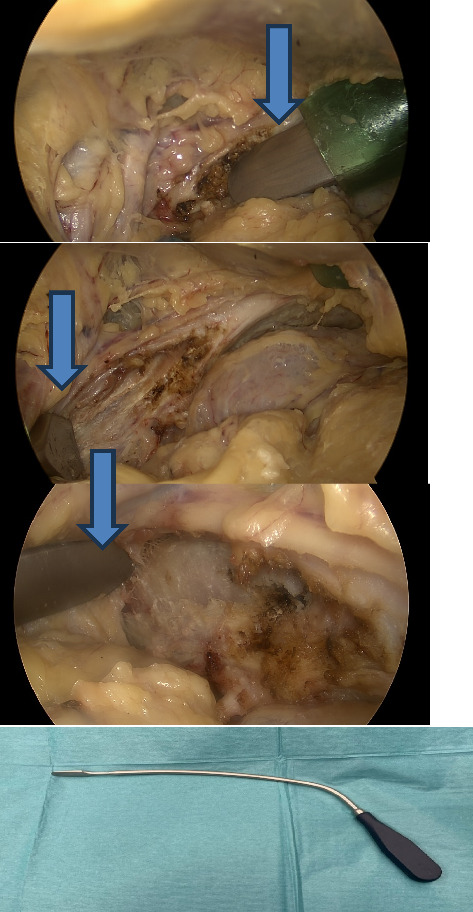
Dissection of the iliopectineal fascia with a curved periost elevator (blue arrow) (Stryker Corporation Kalamazoo, MI, USA).

The remaining tissues are then dissected in the extraperitoneal space and the corona mortis vessels are identified and clipped endoscopically (Endo clip L, Medtronic, MN, USA). The periost is not dissected from the anterior part of the pelvis. The iliopectineal fascia is then dissected and retracted from the muscle area (Figure 2) and the fracture site is revealed. This procedure is performed with a curved periost elevator (Stryker Corporation Kalamazoo, MI, USA) (Figure 2) through ports 2 and 3 in turn. Consequently, the space to the plate is made.

**Figure 3:**
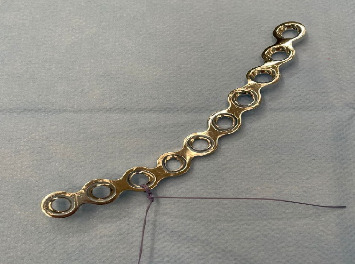
The prebent plate with the braided absorbable suture in the third hole counting from the medial side.

**Figure 4:**
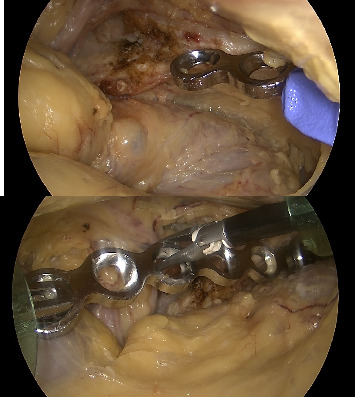
Insertion of the plate into the pelvis using a finger (blue) and forceps.

**Figure 5:**
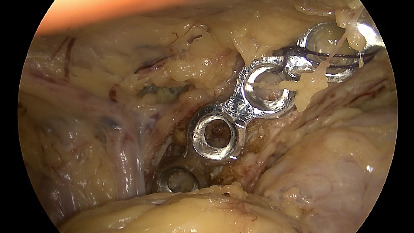
The correct positioning of the plate.

**Figure 6:**
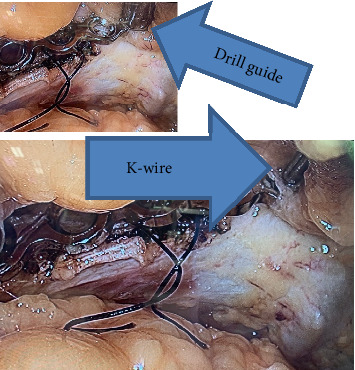
The plate is fixed through a drill guide with a K-wire.

The length of the plate is determined on CT of the patient and bent using the suprapectineal plate (Stryker Corporation Kalamazoo, MI, USA) as a model because it usually fits to the pelvis of everyone quite nicely. We added a braided absorbable suture to the third count hole from the medial side to visualize the correct location of the plate more easily (Figure 3). After that, port 2 is removed and the plate is inserted into the pelvis through the skin incision of port 2 using a finger and forceps (Figure 4). The port 2 is then replaced and the operation is carried out to find the correct position of the plate, which is midline just lateral to the symphysis and runs anterior to the acetabulum and laterally to the pelvic brim (Figure 5). The plate is then temporarily fixed with a K-wire through port 2 using a specific drill guide (Figure 6). The positioning of the plate is then checked with image intensified fluoroscopy.

**Figure 7:**
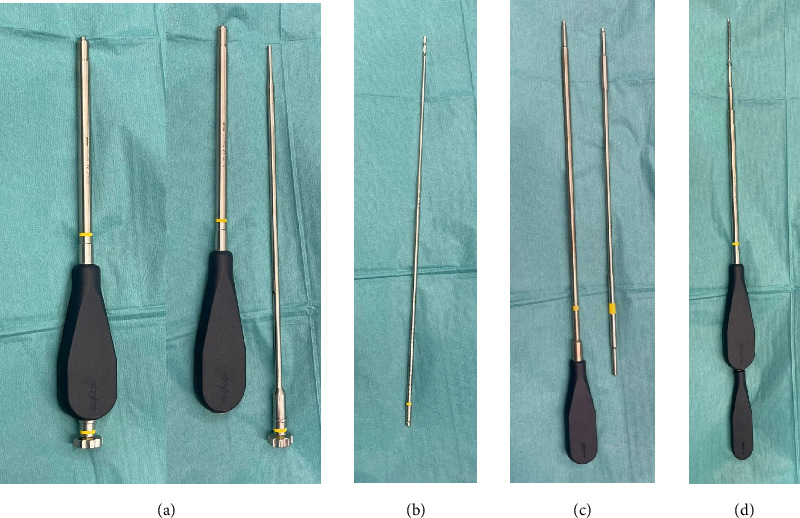
Stryker Pro Matta pelvic drill guide (a), drill (b), screwdriver (c), plate screw inserter together with the screwdriver (d).

**Figure 8:**
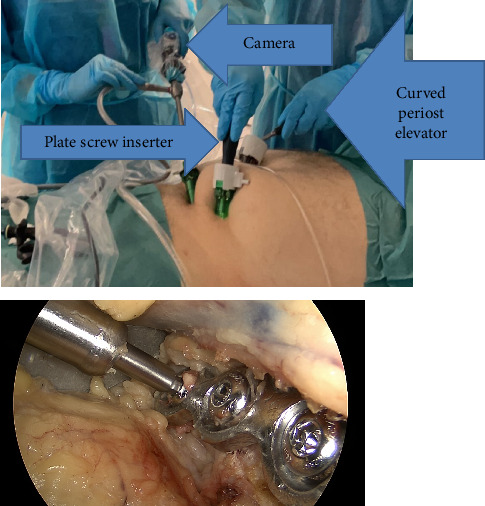
External picture of the ports and instruments and inside picture of the screws.

**Figure 9:**
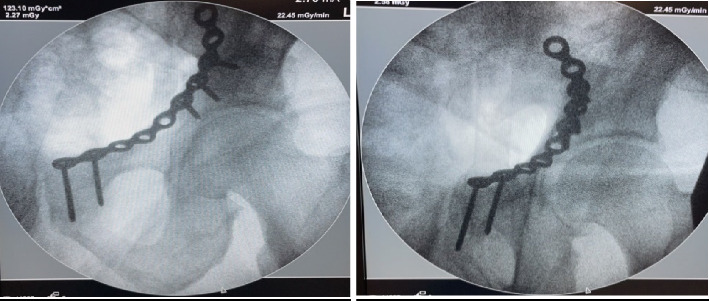
C-arm images of the plate.

The drilling, measuring and screw insertion is performed first through port 3 using a plate screw inserter (Stryker Pro Matta pelvic system, Stryker Corporation Kalamazoo, MI (Michigan)) (Figures 7(a), 7(b), 7(c), and 7(d)). After that, we removed the anterior K-wire and replaced it with a screw so that we could change the camera to port 2. The drilling and insertion of the remaining lateral screws is done through port 3 (Figure 8). We used three screws laterally. The medial screws are likewise inserted through port 2 and the camera is then changed to port 1. Finally, the positioning of the plate and screws is checked with an image intensifier (Figure 9).

The fascia plane of the trocar sites and the skin are closed with sutures (2-0 PDS and Vicryl Rapide, Ethicon NJ; US, respectively).

### 2.2. Fracture Reduction

**Figure 10:**
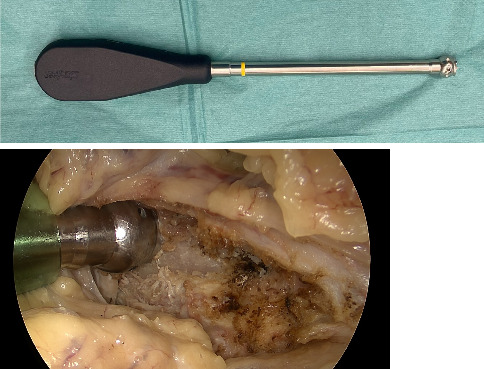
Spiked screw inserter.

Our goal is to test this method in patients and the plan is to reduce the fracture primarily through port 3 with the aid of a ball spike and a spiked screw inserter (Figure 10) with or without a spiked disc. The camera will be in port 1 or port 2, depending on the quality of the visualization. The preliminary fixation of the fracture will be performed by drilling through the inserter, measuring the length of the screw with the help of marks on the drill, and inserting the screw through the inserter. The plate will be inserted when the fracture is fixed first.

## 3. Discussion

It is widely acknowledged that laparoscopic surgery is superior to open laparotomy in terms of wound healing, blood loss, and postoperative problems. Therefore, we began to develop this alternative approach for operating unstable acetabulum fractures [12–14]. This kind of innovative development is made possible by the close relationship between the alimentary tract and orthopaedic surgeons in our hospital and the modern cadaver facilities offered by the Tampere Surgical Education Centre, Finland [15].

The pelvis is a complex structure that includes not only the bony parts but also many nerves, vessels, and soft tissues, which can make it quite challenging to operate an acetabulum fracture. Therefore, we felt that any improvement in surgical technique we could develop would be beneficial to our patients [16]. Additionally, during training in the cadaver laboratory, we found that we could insert screws into the posterior column through the suprapectineal plate with this new method, which is impossible using the modified Stoppa approach. This is a promising finding that means that we can treat patients with fractures of both columns only anteriorly, whereas previously we have operated these through open anterior and posterior approaches.

Our goal in the future is to operate on anterior acetabulum fractures primarily using this endoscopic method. We acknowledge the long learning curve and, therefore, we plan to develop new instruments in cooperation with the implant industry to help working endoscopically in the pelvis [11]. However, with optimized instrumentation, it should be possible to overcome the technical challenges of reducing acetabular fractures and proper positioning of plates and screws. Our findings support the few earlier preliminary reports that a minimally invasive fixation approach in patients with acetabular fracture is feasible [3–5]. Moreover, our results corroborate the earlier experience published by Trulson in four cadavers in 2019 and Hartel in ten cadavers in 2022 [9, 10]. However, in addition to a team of trauma surgeons, it is crucial to have an experienced laparoscopic surgeon with a wide experience of extraperitoneal inguinal hernia surgery involved in these operations.

## 4. Conclusion

A minimally invasive full endoscopic minimally invasive procedure for acetabular fractures offers significant benefits over traditional open surgery due to faster rehabilitation, potentially less blood loss, and fewer wound complications. According to our initial experiences with cadavers, this minimally invasive method appears promising in terms of superior visibility and easier access to the otherwise narrow and difficult fracture site in the pelvic region compared to open surgery. Furthermore, this minimally invasive method seems feasible for exact plate placement in combined endoscopic and fluoroscopic visual control. The usefulness of this novel method in the minimally invasive treatment of acetabular fractures in real life, especially considering the practicality of proper fracture reduction, should be confirmed in future clinical trials.

## Data Availability

The readers can access the data supporting the conclusion of the study by contacting the corresponding author.
